# Immune Responses to the Sexual Stages of *Plasmodium falciparum* Parasites

**DOI:** 10.3389/fimmu.2019.00136

**Published:** 2019-02-11

**Authors:** Jonas A. Kengne-Ouafo, Colin J. Sutherland, Fred N. Binka, Gordon A. Awandare, Britta C. Urban, Bismarck Dinko

**Affiliations:** ^1^West African Centre for Cell Biology of Infectious Pathogens, University of Ghana, Accra, Ghana; ^2^Department of Immunology and Infection, Faculty of Infectious and Tropical Diseases, London School of Hygiene and Tropical Medicine, London, United Kingdom; ^3^Department of Epidemiology and Biostatistics, School of Public Health, University of Health and Allied Sciences, Ho, Ghana; ^4^Faculty of Biological Sciences, Liverpool School of Tropical Medicine, Liverpool, United Kingdom; ^5^Department of Biomedical Sciences, School of Basic and Biomedical Sciences, University of Health and Allied Sciences, Ho, Ghana

**Keywords:** *Plasmodium falciparum*, gametocytes, humoral immunity, cellular immunity, mosquito immunity

## Abstract

Malaria infections remain a serious global health problem in the world, particularly among children and pregnant women in Sub-Saharan Africa. Moreover, malaria control and elimination is hampered by rapid development of resistance by the parasite and the vector to commonly used antimalarial drugs and insecticides, respectively. Therefore, vaccine-based strategies are sorely needed, including those designed to interrupt disease transmission. However, a prerequisite for such a vaccine strategy is the understanding of both the human and vector immune responses to parasite developmental stages involved in parasite transmission in both man and mosquito. Here, we review the naturally acquired humoral and cellular responses to sexual stages of the parasite while in the human host and the *Anopheles* vector. In addition, updates on current anti-gametocyte, anti-gamete, and anti-mosquito transmission blocking vaccines are given. We conclude with our views on some important future directions of research into *P. falciparum* sexual stage immunity relevant to the search for the most appropriate transmission-blocking vaccine.

## Introduction

Malaria is one of the most important parasitic infections with the highest burden of mortality and morbidity in sub-Saharan Africa. Despite progress and advances in the strategies to control the disease, malaria claimed the lives of approximately 445,000 people from among 216 million clinical cases globally in 2016; mostly in children under 5 years and pregnant women as reported by WHO (1). The increasing challenges posed by the emergence of resistance to antimalarials by malaria parasites and to insecticides by mosquitoes (2, 3) suggest the need for additional interventions aiming at transmission reduction such as vaccines. Moreover, targeting of multiple stages of the parasites might be the best strategy for any successful malaria vaccine (4), further highlighting the need for continuous identification and validation of alternative and effective targets.

**Figure 1:**
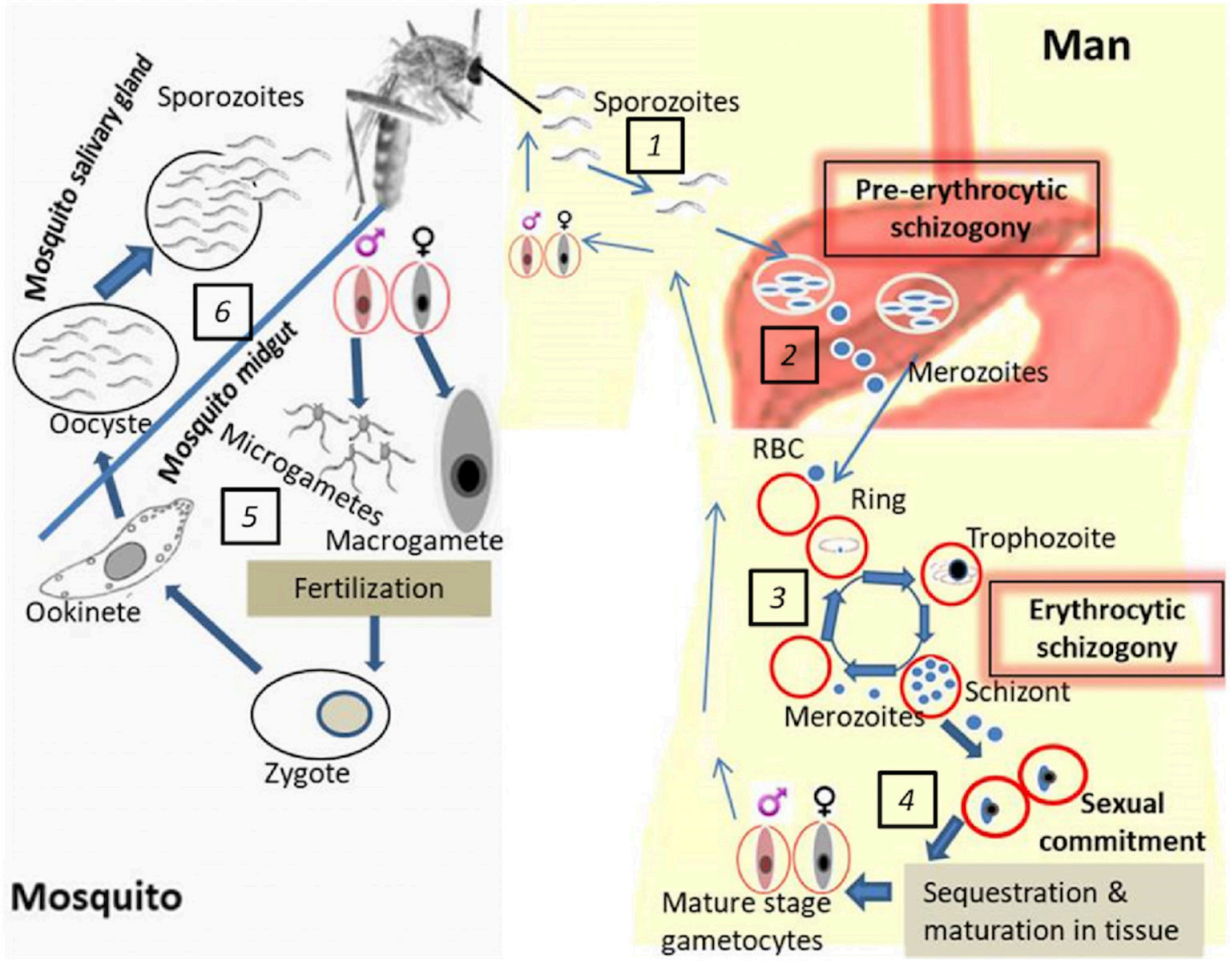
Life cycle of *P. falciparum* development in the human host and mosquito vector. **(1)**. Mosquito's bite and release sporozoites into the human host followed by migration into the liver. **(2)**. Pre-erythrocytic schizogony: infection of hepatocytes and asexual multiplication of the parasites in the liver. **(3)**. Erythrocytic schizogony: translocation of parasites from the liver into the bloodstream accompanied by asexual multiplication and release of merozoites upon RBC rupture. **(4)**. Gametocyte generation: sexual commitment, sequestration of early gametocytes, maturation in tissues and release of mature gametocytes in blood (ready to be picked up by the vector). **(5)**. Parasite development in the mosquito midgut: exflagellation of male gametocytes prior to fertilization which yields the zygote which undergoes further development into a motile ookinete. **(6)**. Parasite development in the mosquito salivary gland: oocyst formation, sporozoite development, and release in the mosquito salivary gland (ready to be transmitted to the human host during subsequent mosquito bites).

Transmission blocking interventions either targeting gametocytes while in the human host or gametes in the mosquito are considered an essential part of malaria control strategies especially in the quest to eradicate malaria (5, 6). Malaria parasites (sporozoites) are transmitted through the bite of Anopheline mosquitoes. Once in the human system, the sporozoites migrate to the liver where they undergo pre-erythrocytic multiplication (schizogony) leading to the production of merozoites that move into the bloodstream (erythrocytic stage; Figure 1). The pathology results from red blood cell (RBCs) invasion and further asexual replication of parasites within RBCs (erythrocytic schizogony) leading to massive RBC lysis, disrupted blood flow due to cytoadherence of parasite-infected RBCs to endothelial surfaces, anemia, and inflammation that may be lethal if untreated. Gametocytes are specialized stages of *Plasmodium* parasites that are essential for transmission from humans to mosquitoes. Initially, a certain proportion of the erythrocytic stage parasites undergoes a permanent differentiation also referred to as sexual commitment into both male (microgametocyte) and female (macrogametocyte) gametocytes (Figure 1). This process is known as gametocytogenesis (7, 8).

Sexually committed ring stage trophozoites from erythrocytic stages in peripheral circulation (9, 10) progress into gametocyte developmental stages 1 to IV while sequestered in bone marrow compartments (11–14). This constitutes the main reason why only late gametocyte stages are found in peripheral circulation. Early gametocytes are thought to sequester in tissues such as spleen and bone marrow through parasite-host interactions via parasite molecules less elucidated but probably PfEMP1, STEVORS, or RIFINS (14–16). The human host endothelial receptors mediating sequestration of developing gametocytes in the bone marrow and other organs however remain unidentified (17). Differentiation of male and female gametocytes occur during sexual commitment where the asexual precursor, schizont, give rise to either male or female gametocytes (7, 8).

After about 10–12 days of sequestered development, mature, male, and female gametocytes emerge and circulate in peripheral blood for a variable amount of time until taken up by mosquitoes (18, 19). Gametocytes do not replicate; however, hemoglobin digestion continues until they reach stage IV (20). In addition, gametocyte-specific mRNAs are produced and a subset of these, important for their stage development in the mosquito, are translationally repressed until gametocytes are taken up by the vector when they go back to peripheral circulation (21). The phenomenon governing the return of mature gametocytes in the peripheral blood is not clearly understood. Once ingested, gametocytes rapidly transform into male (microgamete) and female gametes (macrogamete) in response to environmental cues such as a rise in pH, reduction in temperature and exposure to xanthurenic acid (22). Exflagellation (male gamete induction) is followed by the expression of gamete-specific proteins (23). Fertilization of macrogametocytes by microgametes is preceded by 3 rounds of DNA replication by male gametocytes giving rise to 8 motile microgametes resulting in a zygote (Figure 1). The zygote elongates to form an ookinete which crosses the midgut wall to develop into an oocyst. Further cell divisions and development of the oocyst give rise to sporozoites. Following oocyst capsule rupture, thousands of sporozoites emerge and invade the mosquito salivary glands which then render the vector infectious to humans during a bloodmeal, thus completing the transmission cycle (24–26) (Figure 1).

The infectiousness and transmission potential of gametocytes is influenced by their prevalence and density (27), degree of maturity (28), and both mosquito and human immune responses (29, 30). Furthermore, the efficiency of transmission depends on the generation of sporozoites and therefore level of infectivity or sporozoite dose transmitted (31). Moreover, the sporogonic stages are exposed to the vector's natural immune responses (32–34). It should be pointed out that gametocyte infectiousness refers to the amount of mature gametocytes that can potently infect the mosquito (demonstrated by their ability to undergo further development) after ingestion whereas sporozoite infectivity refers to the dose of potent sporozoites capable of being transmitted to humans during subsequent blood meals.

Here, we review the available evidence for naturally acquired human immune responses against the sexual stages of *Plasmodium* parasites targeting gametocytes and gametes in human and mosquito hosts, respectively. The mosquito immune responses against the development of these sexual stages in the midgut are also discussed, and propositions are made for future research directions toward the design of appropriate transmission blocking vaccines.

## Naturally Acquired Antibody Responses to Gametocyte and Gamete Antigens

For over three decades now there have been some efforts to illuminate antibody responses to gametocyte and gamete development in mosquitoes and their potential for transmission reducing immunity (TRI). TRI is based on observations of naturally acquired antibodies against gametocytes that are produced in the human host in response to proteins of gametocytes that were not taken up by mosquitoes (35). When these gametocytes die, they release intracellular proteins/antigens into the host circulation. Among these are proteins produced in gametocytes which are crucial for the extracellular parasite development in the mosquito midgut (36, 37). These antigens are then processed and presented by antigen presenting cells eventually eliciting humoral immune responses, which can cause substantial or complete blockade of parasite development (gametogenesis, fertilization) in the mosquito. This is the essence of TRI and forms the basis for the development of transmission-blocking vaccines (TBV). TRI occurs when human antibodies, taken up by a mosquito in a potentially infectious blood-meal containing male and female gametocytes, are able to prevent fertilization and/or development of ookinetes/oocyts/sporozoites in the mosquito and thus infection of the mosquito (38, 39).

Extensively studied antigens to date include gametocyte/gamete proteins such as Pfs230 and Pfs45/48 and the zygote/ookinete proteins Pfs25 and Pfs28 (37) also known as the TBV candidate (30, 37, 40–43). Anti-Pfs230 and Pfs48/45 antibodies target the so-called pre-fertilization phase while anti-Pfs25 and anti-Pfs28 antibodies represent the post-fertilization phases marked by the differences in the parasite stage and target antigens. As such parasite proteins are referred to as Pre- and post-fertilization antigens, respectively. Binding of these antibodies to their antigen either blocks their function essential for parasite development or facilitates complement-mediated gamete killing as shown for antibodies against Pfs230 (44).

Naturally occurring antibodies targeting Pfs230 and Pfs45/48 have been observed in field studies in The Gambia, Kenya, and Cameroon and were associated with reduced malaria transmission (30, 45). However, other studies reported that transmission reduction correlated with antibody responses to Pfs230 only (46) or with anti-Pfs48/45 antibodies only (6, 42, 43). These conflicting results may be due to differences in the history of exposure of study participants or existence of other co-infections.

A recent study by Stone et al. using field-based mosquito-feeding assays found mosquito infection rate to be significantly reduced for people harboring naturally acquired anti-Pfs48/45 and anti-Pfs230 antibodies. In addition, these antibodies were shown to be host gametocyte density-dependent and mechanistically associated with transmission reducing activity (TRA) (47, 48). In the same study, using protein microarray, 43 novel gametocyte proteins whose specific antibodies were associated with TRA were also identified (48). Among these 43 proteins, 16 predicted to be surface-expressed showed responses more similar to those of Pfs48/45 and Pfs230 in terms of TRA and as such warrant further investigations and characterization as TBV candidates (48, 49). However, the increase of natural seroprevalence to Pfs48/45 and Pfs230 with age found by Stone and colleagues did not corroborate a previous study by Ouedraogo et al. (50).

It is worth noting that antibodies against the post-fertilization antigens Pfs25 and Pfs28 have not been observed because these antigens are not exposed to the human immune system. If utilized in a vaccine, post-fertilization antibodies would not be boosted by natural malaria infections in vaccinated individuals. Nevertheless, antibodies against Pfs28 and Pfs25 have shown promise in blocking mosquito stage development and therefore transmission in *in vitro* experiments and are currently being evaluated in clinical trials (51).

The development and evaluation of antibody responses to all gametocyte/gamete-specific antigens and their effect on sexual stages in the mosquito face several challenges (51). First, evaluation of transmission reducing immunity relies heavily on mosquito feeding experiments, otherwise known as standard membrane feeding assays, where gametocyte-infected blood is fed to mosquitoes with or without antibodies to the respective antigens. Dissection of mosquitoes 7 days after blood feeding for oocyt counts is used as an indirect measure of transmission blocking activity. These assays are time-consuming and labor-intensive. Second, it is not known what ingested antibody levels in the mosquito are required that would lead to a subsequent transmission blockade. The identification and validation of gametocyte surface antigens as vaccine candidates with transmission reducing activity (TRA) directly measurable in the human host will overcome these challenges, complement and strengthen current transmission blocking vaccine efforts (6, 52).

## Naturally Acquired Antibody Responses to The Surface of Gametocyte-Infected Erythrocytes

It has been difficult to elucidate naturally acquired antibody responses to gametocyte-infected erythrocyte surface antigens (GSAs), distinct from those recognizing internally expressed gametocyte and gamete antigens, in natural human infections. This is largely due to the indirect effect of asexual stage immunity on the prevalence and density of gametocytes.

Naturally acquired sexual stage antibodies are known to be produced against *P. falciparum* gametocyte-infected erythrocyte surface antigens in human peripheral circulation (anti-gametocyte immunity) (4, 53). There are very few studies on human immune responses recognizing gametocyte-infected erythrocyte surface antigens referred to as anti- gametocyte immunity. This is in contrast to anti-gamete immunity which is raised against intracellular proteins of dead gametocytes which have some function at the gamete stages, or more broadly immune responses to gamete surface antigens (4, 6, 31, 52–54).

In the first investigation of its kind, plasma antibodies from gametocytemic Gambian children donated after antimalarial treatment were used to detect antigens on the surface of 3D7 cultured mature stage V gametocytes. Surprisingly, no antibody recognition of the surface of erythrocytes infected with developing gametocytes, stages I-IV, representing the stages known to be sequestered in deep tissues, were found (44, 54). In addition, children harboring these anti-GSA antibody responses were significantly less likely to carry gametocytes after subsequent infections suggesting an ability to control gametocytemia in these patients. It was also shown that malaria patient plasma samples with strong anti-GSA plasma antibody recognition of the mature gametocyte-infected erythrocyte surface were not more likely to recognize the surface of erythrocytes infected with asexual parasites and vice versa (54).

This was a proof of concept for the rationale to develop an anti-GSA transmission blocking vaccine. It derived its basis from epidemiological observations of specific immune suppression of gametocytes in Indonesia (53). *P. falciparum* gametocyte rates were reduced among semi-immune native Papuans, independent of immune control of asexual parasitemia, when compared to a transmigrant Javanese population with a history of lower malaria exposure. These findings suggest specific immune control of gametocytemia as the observations could not be explained by differences in the frequency or grade of parasitemia, illness or by known patterns of antimalarial treatment. Further, immunofluorescence tests with acetone-fixed whole gametocytes showed a correlation between antibody levels and reduced gametocytemia among the native Irianese (53).

The important observations by Saeed et al. (54), the ability of patient plasma to recognize GSA and the significant association with reduced gametocyte carriage, required further investigation. In order to rule out the fact that patient plasma antibody recognition of GSA on mature stage V gametocytes was not a deficiency or artifact of the 3D7 clone, we carried out recognition studies in plasma antibody samples from Ghanaian school children from a high endemicity region, against both a recent isolate and 3D7. In this study, plasma from asymptomatic school children collected over 5 sampling times at weekly intervals were tested against the surface of 3D7 mature gametocyte-infected erythrocytes as well as mature gametocytes derived from a 2012 clinical isolate of Kenyan origin, HL1204. Interestingly, we found plasma antibodies from all children bind to GSA of gametocytes derived from both clones to at least some extent. It was striking to note that plasma from Ghanaian children recognized the GSA on mature gametocytes of Kenyan origin, suggesting that perhaps the antigens detected might be conserved across geographical locations. Immature gametocytes from the clinical isolate were also tested against a selected number of plasma samples from Ghanaian children with strong anti-GSA antibody responses. Similar to the observations of Saeed et al. (54), no detectable recognition of GSA to asynchronous immature gametocytes was observed. These findings were corroborated by some gametocyte adhesion studies (15, 55), which posit that maturing gametocytes do not, as previously thought, sequester from peripheral circulation through adhesion to human bone marrow-derived endothelial surfaces and receptors (56–58). Nevertheless, further studies with tightly synchronized immature gametocyte preparations are required before we can rule out the possibility that developing gametocytes express adhesins involved in parasite ligand-host receptor interactions which mediates sequestration and elicit gametocyte-specific immunity (4).

To further test the prevalence of anti-GSA antibodies in the general endemic population, plasma donated by microscopically-confirmed parasite negative individuals were tested for antibody recognition to GSA. Forty-eight percent (24/50) of parasite-negative children and adults recognized the surface of mature gametocyte-infected erythrocytes (4, 59). Since submicroscopic gametocytemia could not be excluded, anti-GSA antibody carriage in cohort studies utilizing sensitive gametocyte detection methods such as RT-qPCR or QT-NASBA are needed to fully illuminate this relationship. Moreover, testing plasma donated from both gametocyte positive and negative children showed that our findings could possibly represent the general malaria-infected population. In addition, evidence was found that children who harbored anti-GSA antibodies were significantly but weakly associated with lower risk of gametocyte carriage (4). In addition, preliminary indirect evidence suggest that anti-GSA antibodies may be maintained over a period of time (4, 59).

## Cellular Immune Responses to Gametocytes While in The Human Host

Studies aiming at evaluating the cellular immune responses to the sexual stages compared to asexual ones of *Plasmodium* species are limited. However, there is evidence that such immunity exists. The transfer of T-cells from gamete-immunized mice was shown in the 1980s to markedly reduce gametocytemia in the recipient mice using the rodent species *P. yoelii nigeriensis* (60). Recipient mice also failed to effectively infect the mosquito vector *A. stephensi* as demonstrated by direct blood feeding (up to 95% transmission reduction) suggesting the direct impact of T-cells on transmission. However, the T-cell transfer had no effects on asexual stages (60). Good et al. (61) further showed that peripheral blood from non-exposed individuals contains T cells (clone) which proliferate and up-regulate interferon-gamma production upon stimulation with mature gametocyte-infected RBCs lysate. Similar results were also obtained in a hyper-endemic region in The Gambia by stimulation of peripheral blood mononuclear cells (PBMCs) of volunteers by gametocyte lysate (62). The detection of gametocyte-specific antibodies in the study participants by ELISA implied previous exposure to sexual stage parasites. Findings from this study suggest a T cell-dependent suppression of gametocytes or T cells helping B cells to fight against the malaria infection. Both asexual and sexual stage-specific antigens have equally been shown to elicit polyclonal T-cell responses in malaria non-exposed individuals (63). However, the reaction is not peculiar to gametocytes since it has been demonstrated that CD4 T Cells from non-exposed individuals react with PfEMP-1 via a Major Histocompatibility Complex (MHC) Class II-T cell receptor-independent Pathway (64). This could be associated to cross-reactivity from other infections. Whether this phenomenon is protective in children is not known.

In other studies, an increase in cytokine production such as TNF-α and IFN-γ was demonstrated in monkeys and humans infected with *P. cynomolgi* and *P. vivax*, respectively (65, 66). The increase in cytokine secretion correlated with the decrease in parasitemia and the inability of gametocytes to infect the mosquito vector and as such, cytokines and other PMBC-derived components (nitric oxide, antibodies) were believed to play a role in the loss of infectivity (66, 67). In their study in 1993, Naotunne et al. showed that the effect of PBMC-derived components on gametocyte infectivity was closely linked to the presence of white blood cells as no effect was seen in their absence (67). This negative effect on the infectivity of gametocytes appeared to be reversed in the presence of high concentration of an L-arginine analog (NGL-monomethyl arginine acetate) (68). This suggests that gametocyte inactivation in the presence of WBCs is achieved through an L-arginine-associated pathway mechanism. In the same line, an *in vitro* study conducted by Smith et al. (69) revealed that *P. falciparum* stage I and IIA gametocytes are to a large extent eliminated from the circulation by non-opsonic phagocytosis mediated by monocytes and macrophages. They showed through antibody inhibition assays and enzyme treatment that the interaction of PfEMP-1 and CD36 plays a major role in this innate defense against early gametocytes stages. This is in line with a previous study reporting the interaction of *P. falciparum* early gametocytes with CD36 receptor (70). A recent study conducted in India demonstrated a significant negative association between gametocytemia and IFN-γ in children (71).

Gametocyte-specific exoantigens (from gametocyte culture supernatants) have been shown to be able to stimulate the proliferation and activation of lymphocytes from *P. falciparum* exposed individual (72). In this study, T cell receptors gamma/delta (TCR γδ+), and CD3^+^ CD8^+^ and CD3^+^ CD4^−^ CD8^−^ T cells were found to be up-regulated upon sensitization with these exoantigens. Particularly, the expression of the activation marker CD25^+^ increased on stimulated CD3^+^ and γδ T cells. The frequency of γδ T cells had previously been found to increase in the course of acute malaria (73). However, it is difficult to ascertain the specificity of the exoantigens because they could as well be coming from ruptured or dead asexual parasite iRBCs. As already indicated, there is a paucity of information on cellular immunity to gametocytes. Therefore, further investigations into the role of cellular immune responses to gametocytes and malaria transmission; and the identification/validation of the antigens involved, in a bid to contribute toward the development of an effective transmission blocking vaccine are required.

## Cellular Immunity to Sexual Stages While in the Mosquito Vector

In the mosquito vector, killing of malaria parasites is not only mediated by vertebrate host-derived molecules but also by mosquito components as has previously been demonstrated (32–34). Studies have shown that only a small proportion of gametocytes ingested in the blood meal by the mosquito vector is transformed into oocysts and sporozoites; and only about 38% of mosquitoes that take gametocyte-containing blood become infected (32, 33, 74). This is largely due to the peritrophic membrane or matrix (PM) which constitutes a physical barrier to *Plasmodium species* and other pathogens (33, 75, 76). This membrane is formed after the ingestion of a potentially infectious blood meal by the mosquito and surrounds the ingested blood. It prevents direct contact between the pathogens in the blood and the midgut epithelium and by so doing interferes with midgut invasion (33, 76). However, ookinetes secrete the enzyme chitinase which destroys the chitineous PM and allows it to invade the midgut (33, 77). The midgut epithelial cells are also thought to secrete high amounts of nitric oxide synthase and peroxidases, which in turn leads to nitration of the gut epithelium with subsequent tagging of ookinetes for destruction by the complement system (33, 78, 79).

The innate immune response in the malaria mosquito vector is mediated mainly by hemocytes which eliminate pathogens such as bacteria, fungi, and protozoa by phagocytosis (80–82). The *Anopheles* species and other insects are known to have a complement C3-like protein called thioester-containing proteins (TEP) (80). TEP of *A. gambiae* (AgTEP1) has been shown to be valuable for the initiation of immune defense against *P. berghei*. TEP1 plays the role of opsonins and facilitates the interaction between the parasite and the hemocytes with subsequent encapsulation, and killing of the parasite (80). Double knock-out of the TEP1 gene renders genetically selected refractory *Anopheles* strain susceptible to infection and increases the infectivity rates in susceptible *A. gambiae* (80). This vector defense mechanism has been shown recently to be by-passed by *P. falciparum* through its 6-cysteine protein P47-like (83). This protein is invaluable for *P. berghei* female gamete fertility (84) but in *P. falciparum*, it promotes the gametocyte-to-ookinete development and protects the ookinete from complement-dependent lysis (83).

In addition, infection of *A. gambiae* mosquito by ookinetes of *P. berghei* has been demonstrated to modulate the mosquito's immune system by up-regulating the expression of the antibacterial peptide defensin and a putative gram-negative bacteria-binding protein (85), and a TNF-α factor-like transcription factor (LL3) (86). Silencing of the LL3 gene was found to be associated with an increase in parasite survival, confirming its role in conferring mosquito resistance to the *Plasmodium* parasite. LL3 also affects the expression of another protein, a serine protease inhibitor (SRPN6), which equally confers resistance to invasion by *Plasmodium* (86).

Genomic and transcriptomic analyses of bacterial lipopolysaccharide-stimulated *A. gambiae* mosquitos revealed 23 immune-regulated genes which include putative protease inhibitors, serine proteases, and regulatory molecules (87). Interestingly, the protease inhibitor α-2-macroglobulin was found to be more specific in response to malaria parasite than bacterial infection as observed with mosquitoes fed on a *P. berghei*-infected hamster. This suggests that the immune response mounted by the mosquito vector may be pathogen specific, and other authors have reported similar findings (88). RNA gene interference (RNAi) experiments on *P. falciparum-* and *P. berghei-*infected *An. gambiae* revealed some common genes that confer resistance to both parasite species. However, other genes were found to exhibit species-specificity, conferring resistance only to one parasite species, namely a pattern recognition receptor (MD2-like receptor, AgMDL1) and an immunolectin, FBN39 for *P. falciparum* and the antimicrobial peptide gambicin and a novel putative short secreted peptide, IRSP5 for *P. berghei* (88). Together, these findings show that mosquitoes express molecules with anti-plasmodial properties which act as self-defense mechanism in the vector.

## Anti-gametocyte and Anti-gamete Transmission Blocking Vaccines

Up to date, it has been a difficult task developing an effective vaccine against malaria. This is due both to the complexity of the *Plasmodium* parasite life cycle and the polymorphic nature of its antigens (89, 90). However, the hope that an effective malaria vaccine is feasible is based on the observation that in endemic regions, clinically immune adults are protected from severe malaria and death compared to children (89). This could be attributed to the fact that natural immunity in adults is probably complex and dependent on immune responses to many stages. Interestingly, sera from immune individuals have been shown to inhibit gamete fertilization and development in the mosquito vector thereby interfering with disease transmission (91–93). This constitutes the basis of the development of malaria transmission blocking vaccines (TBVs). An emerging concept is to develop vaccines against antigens expressed solely in the mosquito's midgut to which the host immune system is not naturally exposed. Antibodies against those antigens from vaccinated individuals and animals have been shown to interfere with parasite viability and development in the mosquito midgut interaction (94–96).

**Figure 2:**
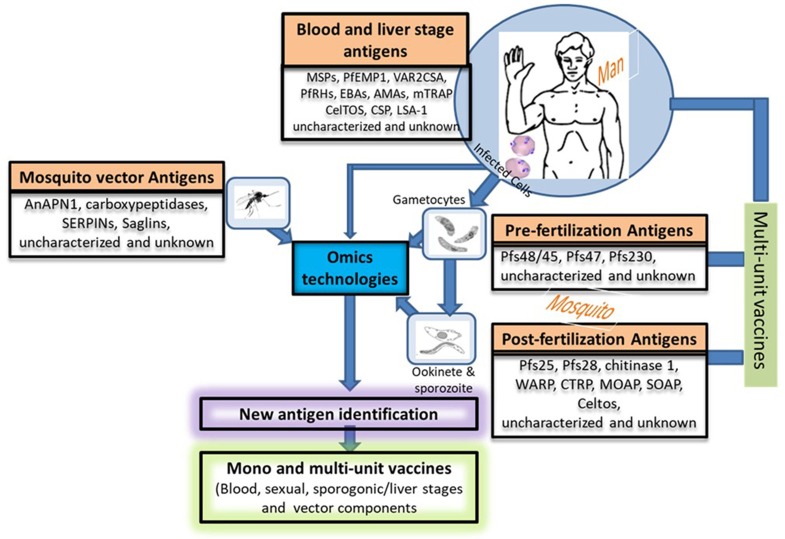
Potential approaches for new vaccine candidate identification and vaccine development. This figure describes in a nutshell the life cycle of the *Plasmodium* species providing different stage-specific antigens and the strategies that could be used to develop more efficacious vaccine by combining potential vaccine candidates from various stages (host's and vector's). Uncharacterized and unknown antigens refer to proteins of unknown functions or proteins that have only been partially characterized such as the multigene families (*var, rif, and stevor*). The function of the proteins encoded by these multigene families have been tremendously studied in the asexual stages of *Plasmodium* but poorly exploited in their sexual counterparts. We propose that more attention should be paid to the sexual stages in terms of vaccine candidate identification and characterization. Nowadays, this could easily be done with the advent of Omics technologies. PfEMP-1, *Plasmodium falciparum* erythrocyte membrane protein-1; RIFINs, repetitive interspersed families of polypeptides; STEVORs, Subtelomeric variable open reading frame polypeptides; MSPs, Merozoite surface proteins; VAR2CSA, A variant of PfEMP-1 with high affinity to placental tissues; PfRHs, *Plasmodium falciparum* reticulocyte binding protein homologs; EBAs, Erythrocyte binding antigens; AMAs, Apical membrane antigens; CSP, Circumsporozoite protein; LSA-1, Liver-Stage Antigen; mTRAP, Merozoite thrombospondin-related anonymous protein; Pfs (230, 48/45, 25,28), *Plasmodium falciparum* gamete surface proteins (molecular weight in dalton); WARP, von Willebrand factor-A domain-related protein; CTRP, Circumsporozoite and TRAP-related protein; MAOP, Membrane-attack ookinete protein; SOAP, Secreted ookinete adhesive protein; CelTos, Cell-traversal protein for ookinetes and sporozoites; AnAPN1, *An. Gambiae* alanyl aminopeptidase; serpins, Serine protease inhibitors; *saglin* proteins.

As a limitation, TBVs are different from the other vaccine types (liver and blood stage vaccines) in the sense that they do not protect against disease in the vaccinees. However, they reduce the risk of transmission to other people by the mosquito vector and by so doing favor herd immunity; as such they have sometimes been referred to as altruistic vaccines (95). Two groups of target antigens (gene superfamilies) exist, namely pre-fertilization and post-fertilization antigens (Table 1 and Figure 2) (48, 97, 107–111, 121). The list (Table 1) is not exhaustive both for pre- and post-fertilization antigens as some proteins remain unidentified to date. Some of these target antigens were characterized back in 1983 by Kaushal et al. (98), among them, Pfs48/45, Pfs47, Pfs230, and Pfs25 are immunogenic and less polymorphic, making them good vaccine candidates (122). Their use in combination with strong adjuvants or carrier proteins has been shown to boost their immunogenicity. Some of the adjuvants/carrier proteins used included Maltose Binding Protein (MBP)—Exoprotein A (EPA) from *P. aeruginosa—*Outer Membrane Protein Complex (OMPC)—modified Lickenase carrier (LiKM)—Virus-like particle (VLP)—Alhydrogel. Only two of these vaccine candidates, namely Pfs 230 and Pfs25, have entered clinical trial stage (123) and are reviewed in this paper. The potential of the other antigens (e.g., Pfs48/45) as TBV candidates has been recently reviewed by Chaturvedi et al. (122). Although Pfs 45/48 has not yet attained the clinical trial phase, previous studies demonstrated that antibodies against this antigen elicit up to 99% inhibition of oocyst intensity and 85% inhibition of oocyst prevalence (99). Hence the necessity to pursue studies with the Pfs 48/45 TBV candidate. Moreover, recent studies have identified new sexual stage antigens that require more attention (48, 121)

**Table 1:** Sexual stage antigens in the human host and mosquito vector with transmission reducing activity/potentials.

**Type**	**Antigen name**	**Function/role**	**References**
Parasite pre-fertilization antigens	Pfs48/45	Male gamete attachment to female gamete	(6, 30, 42, 43, 97, 98)
	Pfs47	Fertilization process	(97, 98)
	Pfs230	Main component in the male fertilization process	(30, 41, 98–101)
	STEVORS	Sequestration of early gametocytes and deformability of mature gametocytes	(14–16)
	*Plasmodium falciparum* surface related antigen (PfSRA)	Erythrocyte invasion, unknown role in gametocytes	(102)
	*Plasmodium falciparum* LCCL domain-containing protein (CCP)	Parasite development in the mosquito	(103)
	*Plasmodium falciparum* CX3CL1-binding protein 2	Cytoadherence to host cells	(104)
	*Plasmodium falciparum* Gametocyte EXported Protein-5 (PfGEXP5)	Gametocyte switching	(105)
Parasite post-fertilization antigens	Pf25	Parasite survival and interactions with mosquito midgut	(37, 51, 98)
	Pfs28	Parasite survival and interactions with mosquito midgut	(37, 51, 98)
	Chitinase 1	Parasite invasion of the midgut	(33, 77, 106)
	Von Willebrand factor-A domain-related protein (WARP)	Ookinete attachment to the mosquito midgut, differentiation of ookinete to oocyst	(107)
	Circumsporozoite and thrombospondin-related anonymous protein (CTRP)	Transition from ookinetes into oocysts in the vector	(108)
	Membrane-attack ookinete protein (MAOP)	Ookinete midgut invasion in vector	(109)
	Secreted ookinete adhesive protein (SOAP)	Ookinete midgut invasion and oocyst development	(110)
	Cell-traversal protein for ookinetes and sporozoites (CelTOS)	Establishment of malaria infections in both vector and vertebrate hosts	(111)
Vector antigens	Midgut-specific alanyl aminopeptidase (AnAPN1)	Ookinete midgut invasion in vector	(75, 112–115)
	Carboxypeptidase B1	Parasite development in the vector	(116, 117)
	Serine protease inhibitors (serpins)	Regulation of the vector innate immune responses	(118)
	Saglin proteins	Vector salivary gland invasion	(119, 120)

Pfs230 is a 363 kDa protein and a potent antigen of malaria TBV. It is a main component in the fertilization process as male gametes with impaired Pfs230 gene are incapable of interacting with red blood cells (RBCs) and forming exflagellation centers (100). This results in marked reduction in oocyst production and mosquito infectivity. Similar observations were made with genetically modified *P. falciparum* with a truncated chitinase 1 (PfCHT1) gene which could be due to the inability of affected ookinetes to invade the mosquito midgut (106). Administration of recombinant Pfs230 + Alhydrogel induced high titers of antibodies in rabbits which were found to have significant transmission reducing activity (101). It should be pointed out that only certain fragments of the recombinant Pfs230 antigen induce responses that lead to TRA (99). Significant associations between suppression of mosquito infectivity and anti-Pfs230 antibody levels were also found using membrane feeding assays with sera collected from African populations (43, 45, 46, 124) and in mice (99). This vaccine candidate (125) has entered a phase 1 clinical trial in which the Safety and Immunogenicity of Pfs230D1M-EPA/Alhydrogel is being evaluated in adults in the US and Mali (data unpublished, https://clinicaltrials.gov/ct2/show/NCT02334462).

Pf25 is relevant for parasite survival and interactions with mosquito midgut molecules prior to invasion. Anti-Pf25 antibodies have been shown to halt parasite growth within the mosquito in membrane feeding assays as reviewed by Chaturvedi et al. (122). This vaccine candidate has undergone clinical trial phase 1 in combination with different carriers and adjuvants. Administration of Pf25-Viral-like-particles plus Alhydrogel® to mice resulted in high antibody titers with 100% transmission reducing activity (TRA) throughout the study (126). A clinical trial phase 1a with this combination was recently carried out in the United States (https://clinicaltrials.gov/ct2/show/NCT02013687). It appeared that the combination is safe with no serious adverse reactions observed in healthy volunteers even when higher doses are administered. However, the antibodies generated showed low TRA hence the necessity to prioritize vaccine adjuvant formulations for further investigations (127).

Another combination of Pfs25 and EPA (Pseudomonas aeruginosa ExoProtein A) plus Alhydrogel® was also demonstrated to be well tolerated by naïve individuals after several doses in a phase 1a dose-response clinical trial in the US which correlated with antibody titers (128). A Phase 1b trial of Pfs25-EPA/Alhydrogel® is currently ongoing in Malian adults (122). New promising multimeric Pf25-based Vaccine Candidates ChAd63 Pfs25-IMX313 and MVA Pfs25-IMX313 have recently been developed with promising results (129) and are now undergoing clinical trial phase 1a in the UK. These vaccine candidates consist of attenuated viruses (ChAd63-chimpanzee adenovirus 63 and MVA- modified vaccinia Ankara) encoding the parasite protein Pf25, which are fused to a carrier protein (IMX313-multimerization technology) as adjuvant (NCT02532049). In mouse models, Chad63Pfs25-IMX313 was safe and significantly more immunogenic with higher TRA than monomeric Pfs25 (130). Similar results were obtained with the *P. vivax* antigen (Pvs25H/Alhydrogel), and the *P. falciparum* ortholog Pfs25 in a mouse model. Anti-Pvs25H antibody levels peaked after the third vaccination and vaccine-induced antibodies were functional, giving significant TRA (96). This combination has proven to be safe in humans in a clinical trial phase 1 with similar immunogenicity and TRA as previously shown in mice (131).

The main limitations of these sexual stage vaccine candidates have been the systemic reactogenicity observed in some clinical trials, short-lasting antibody responses owing to the fact that the host has no or limited exposure to the antigens requiring multiple boosting doses and strong adjuvants. In addition the recombinant antigens are difficult to express in their native form (122). To circumvent the issue of limited exposure of pre-fertilization and post-fertilization antigens to the human immune system, it might be good to develop DNA or viral vector-based vaccines containing different antigens (132, 133). This ensures continuous production of the antigens of interest in the host hence permanent stimulation of the immune system, thus bypassing the necessity for multiple immunizations (132, 133). However, the main problem is that the attenuated virus used can revert and cause infections, it can also get integrated in the vaccinee's genome and lead to unforeseen consequences with the associated ethical issues. It would be valuable to make use of high class adjuvants such as the Polymeric nanoparticle- and microparticle-based adjuvant systems that ensure long term delivery of the antigen to the host system when administered (134). It would be good also to include some blood and liver stage antigens to such combinations so that the vaccinee benefits from the process (Figure 2).

## Anti-mosquito Transmission Blocking Vaccines

Some mosquito components are invaluable for the sporogonic development of malaria parasites as they are involved in parasite invasion through interaction with parasite receptors. Antibodies raised against these components could be very useful in blocking parasite development in the mosquito vector (95). Thus, these components constitute another class of TBV candidates as their inhibition would likely minimize the risk of new infection in the community (135). An alternative transmission blocking vaccine strategy could be to interfere with the interactions between the parasite and the midgut molecules of the mosquito vector which will lead to the inhibition of ookinete invasion and the development of mosquito stages. Many of such molecules have been identified and characterized (Table 1 and Figure 2). These molecules have been shown to be more conserved than the parasite antigens and are immunogenic in non-human primates (75, 112–115).

Midgut-specific alanyl aminopeptidase (AnAPN1) is a Glycosylphosphatidyl inositol (GPI)-anchored antigen which plays a valuable role in ookinete invasion in *A. gambiae* as previously shown by Dinglasan et al. (113). Administration of a recombinant fragment of rAnAPN160–195 with Alhydrogel has been demonstrated to stimulate a sustained production of antibodies with transmission blocking activities as revealed by membrane feeding assays (112). The TRA was dose dependent with higher antibody levels attaining 100% efficacy, and functional in both the chromosomal M and S forms of *A. gambiae* vectors. Moreover, the *P. falciparum*-infected blood samples used for membrane feeding assays were collected directly from gametocyte positive individuals and the results obtained exceeded those that had been reported previously with laboratory strains (113). More importantly, anti rAnAPN160–195 antibodies had effects on both *P. falciparum* and *P. vivax* and based on that evidence the AnAPN1 TBV has been recommended for phase I clinical trials (112). This antigen is immunogenic in mice and rabbits even in the absence of adjuvants (113, 115). However, it is worth mentioning that another study by Kapulu et al. failed to replicate the finding reported here. In their study, anti-AgAPN1 IgG had no significant impact on oocyst prevalence (99). Antibodies to another GPI-anchored vector midgut protein, α-AgSGU, were also confirmed to have an effect on *P. falciparum* and *P. vivax* development in *An. gambiae* and *An. Dirus* (114). However, high doses of α-AgSGU antibodies were required to achieve 80% TRA rendering α*-AgSGU* less promising as a TBV target.

In the same line, the midgut carboxypeptidase gene of *A. gambiae* (cpbAg) has been shown to be up-regulated following *P. falciparum* gametocyte ingestion by the vector (116). In addition, anti-CPBAG antibodies were shown to inhibit the development of both *P. falciparum and P. berghei* in the vector's midgut. Antibodies directed against CPBA have also been demonstrated to be vector-unspecific in the sense that they also inhibit the development of the *P. falciparum* gametocytes in *A. stephensi* mosquitoes, which is the main malaria vector in Iran and neighboring countries (117). This confirmed the conserved nature of molecules across different vector as predicted using genomic and proteomic approaches (117) and implies that a vaccine designed with CPBA could provide cross-species protection. Thus, CPBA constitutes another promising TBV candidate.

The interaction between the *Plasmodium* sporozoite Thrombospondin Related Anonymous Protein (TRAP) and the mosquito Saglin proteins is a prerequisite for vector salivary gland invasion (119). This has been confirmed by *in vivo* down regulation experiments of *saglin* gene expression which revealed a negative association with salivary gland invasion (119). Moreover, *in silico* analysis of *saglin* revealed the presence of a signal peptide suggesting that it may be a secreted protein. If verified *in vitro* and *in vivo*, Saglin proteins could constitute a new promising candidate for TBV design (120). Similarly, RNA interference silencing and knock-down experiments have demonstrated the essentiality of the serine protease inhibitors (serpins) in the survival of *An. gambiae* and *An. stephensi* as well as in the development of parasites (*P. berghei*) within these vectors (136, 137). Serpins are regulators of the vector innate immune responses and they are involved in the clearance of protozoan parasites (137). Antibodies raised against the *An. gambiae* serpin-2 (AgSRPN2) have been shown to be *P. berghei*-specific in *An. gambiae* and *An. stephensi* as they failed to interfere with *P. falciparum* oocyst formation (118). This study demonstrated that mosquito innate immune response-related molecules could be used as targets for TBV design; however, further investigations are needed to identify and/or validate the right antigens. A limitation here will be that all of these proteins are likely to suffer from the same problems as gamete/ookinete antigens in the sense that several booster doses are required and antibodies may be short-lived.

## Future Directions in Sexual Stage Immunity and Vaccine Development

Apart from studies reported in these reviews (60–62, 69, 72, 73), studies on host cellular and humoral immunity to gametocytes are scarce if not inexistent. Given promising results in clinical trials of TBV experimental vaccines for malaria eradication, the antigens involved should be characterized further to explore their suitability as vaccine candidates. The generation of long-lived antibodies depends on the generation of long-lived plasma cells and memory B cells (MBCs) within germinal centers (GCs) of secondary lymphoid organs (138). The prerequisite for plasma cells and MBCs is the interaction between follicular T helper cells and B cells. Further investigations of these cell types vis-à-vis the identified antigens in the context of malaria infections are needed. Similarly, further studies aiming at identifying new antigens (mainly vector-, gametocyte-, ookinete-, and/or oocyste-related) using genomics, transcriptomics and proteomics approaches, the Sanger center parasite gene knock-out library and other bioinformatics strategies are warranted (139–141) (Figure 2). These approaches take advantage of next generation sequencing (NGS) and the availability of growing numbers of *P. falciparum* whole genome sequences to identify new antigens (142). These methods are relatively fast and high throughput, leading to the identification of a plethora of essential genes or antigens through comparative analyses (139, 141–143). The implications of omics in the fight against infectious disease was recently reviewed by Bah et al. (144). But this would be strengthened by the concomitant ability to cultivate the sequenced lines and generate sexual stages from them for phenotypic studies. Bioinformatic strategies can also overcome some of the difficulties in studying parasites such as *Plasmodium spp*. or *Trypanosoma spp*. which are genetically diverse (140, 145–147). In addition, computer-based algorithms have been developed to delineate T-cell epitopes on essential parasite proteins directly from genome sequence data (148–150). Vaccine developers should consider designing multi-unit or multi-stage TBVs with components from both the parasite (precisely gametocyte antigens) and the vector as this will broaden their spectrum of action.

As far as the search for a vaccine is concerned, much has been done to understand the multigene family PfEMP-1. However, the other multigene family proteins such as RIFIN and STEVOR also constitute an important class of parasite molecules that deserve attention. These form part of the uncharacterized or partially characterized parasite antigen repertoire with respect to the sexual stages (Figure 2). The *stevor* multicopy family is made up of a set of 39 genes with 2–3 copies expressed at a time (151) while about 150–200 genes code for RIFINs with many copies expressed at a time as well (152). STEVOR and RIFIN proteins were recently shown to be implicated in rosetting which is a phenomenon associated with sequestration and clinical complications of the malaria (153–157). STEVORs are suspected to be implicated in the sequestration of early gametocytes in tissues such as the bone marrow and spleen as well as the deformability of the mature gametocytes (14–16). There is also evidence that STEVORs alter RBC membrane rigidity since RBC deformability has been shown to be linked to STEVOR dissociation from the mature gametocyte-infected RBC membrane (15). This implies that inhibiting the functions of STEVORS might negatively affect the development of gametocytes and by so-doing will also reduce disease transmission due to a reduced number of sexual stages being ingested by the mosquito vector during its blood meal. Despite their variable nature, the putative role of STEVORs in gametocyte development and sequestration certainly make this family a possible new class of TBV vaccine targets. Humoral responses to these antigens have been demonstrated (158). We therefore recommend further characterization (both humoral and cellular) of anti-STEVOR immune response, in the hope of finding additional clues in the search for efficient TBVs. However, the most pressing task is to develop further STEVOR-specific reagents demonstrating their relevance in anti-gametocyte immune responses and therefore transmission reducing immunity/activities.

In addition to STEVORS, many other Plasmodium antigens such as LCCL domain-containing protein family (103), *Plasmodium falciparum* Surface Related Antigen (PfSRA) (102), CX3CL1-binding protein 2 (104), Gametocyte EXported Protein-5 (PfGEXP5) (105) have been described as potential TBV candidates and as such deserve further characterizations.

## Author Contributions

JK-O and BD designed and drafted the manuscript. CS, FB, GA, and BU reviewed and edited the manuscript. All authors approved the final version of manuscript for publication.

### Conflict of Interest Statement

The authors declare that the research was conducted in the absence of any commercial or financial relationships that could be construed as a potential conflict of interest.
